# Systematic Identification of Determinants for Single-Strand Annealing-Mediated Deletion Formation in *Saccharomyces cerevisiae*

**DOI:** 10.1534/g3.117.300165

**Published:** 2017-08-17

**Authors:** Maia Segura-Wang, Megumi Onishi-Seebacher, Adrian M. Stütz, Balca R. Mardin, Jan O. Korbel

**Affiliations:** European Molecular Biology Laboratory, Genome Biology Unit, 69117 Heidelberg, Germany

**Keywords:** *Saccharomyces cerevisiae*, structural variants, single-strand annealing, deletion formation

## Abstract

To ensure genomic integrity, living organisms have evolved diverse molecular processes for sensing and repairing damaged DNA. If improperly repaired, DNA damage can give rise to different types of mutations, an important class of which are genomic structural variants (SVs). In spite of their importance for phenotypic variation and genome evolution, potential contributors to SV formation in *Saccharomyces cerevisiae* (budding yeast), a highly tractable model organism, are not fully recognized. Here, we developed and applied a genome-wide assay to identify yeast gene knockout mutants associated with *de novo* deletion formation, in particular single-strand annealing (SSA)-mediated deletion formation, in a systematic manner. In addition to genes previously linked to genome instability, our approach implicates novel genes involved in chromatin remodeling and meiosis in affecting the rate of SSA-mediated deletion formation in the presence or absence of stress conditions induced by DNA-damaging agents. We closely examined two candidate genes, the chromatin remodeling gene *IOC4* and the meiosis-related gene *MSH4*, which when knocked-out resulted in gene expression alterations affecting genes involved in cell division and chromosome organization, as well as DNA repair and recombination, respectively. Our high-throughput approach facilitates the systematic identification of processes linked to the formation of a major class of genetic variation.

Single nucleotide variants (SNVs) and genomic SVs can be caused by defects in DNA repair systems that are conserved across species. Consequently, they can have a significant impact on phenotypic variation and evolution (Zhang *et al.* 2009; Stankiewicz and Lupski 2010), and are the underlying basis of various diseases (Branzei and Foiani 2010; Carvalho *et al.* 2010). In the budding yeast *Saccharomyces cerevisiae*, spontaneous large chromosomal rearrangements (of ≥ 500 bp in size) resulting in focal deletions or duplications occur at relatively low rates, owing to high selective pressures in the context of the relatively small and compact yeast genome. For example, Zhu *et al.* (2014) followed 145 diploid yeast mutation accumulation lines during > 2000 generations, identifying three large copy number variants of a size > 50 bp when compared to 867 SNVs and 26 indels < 50 bp in size.

These results have initially suggested a relatively high stability of the yeast genome. However, rates of SV formation can be increased by disruption of the pathways involved in DNA repair, recombination, and replication (Myung *et al.* 2001a; Kolodner *et al.* 2002; Smith *et al.* 2004; Kanellis *et al.* 2007). In particular, the SSA repair pathway of homologous recombination can be highly mutagenic due to the use of homologous repeats for DNA double-strand break repair leading to deletions between the repeats (Bhargava *et al.* 2016). Several genes in these repair pathways have human homologs mutated in cancer and in cancer susceptibility syndromes. For example, the knockout of the DNA helicase *SGS1* gene in yeast, homolog to the human *BLM* gene, shows hyper-recombination and genomic instability resembling the characteristics of Bloom’s syndrome patients (Ellis *et al.* 1995; Watt and Hickson 1996). Although several individual examples have revealed a number of genes that might regulate SV or SNV formation, a comprehensive analysis of genes facilitating genomic stability through preventing deletion formation has thus far been lacking.

In addition, when comparing genomes of yeast strains, the total number of base pairs affected by structural rearrangements surpass those affected by alterations of single nucleotides (Carreto *et al.* 2008; Serero *et al.* 2014), implicating SVs as a major class of genetic variation relevant in yeast. Identification of all genes involved in the formation of these alterations will thus be very important not only for understanding principles of evolution in yeast but also for human disease.

Here, we describe the development and application of a genome-wide assay in order to identify budding yeast knockout mutants prone to SV formation, specifically *de novo* formation of intermediate-sized deletions (between 400 bp and 1 kb), an SV class thought to be highly relevant for evolution, population diversity, and disease (Schacherer *et al.* 2009; Weischenfeldt *et al.* 2013; Sudmant *et al.* 2015). In our assay, yeast knockout strains exhibiting increased SSA-mediated deletion formation are identified by screening mutants in a pooled *S. cerevisiae* gene deletion library. Application of our assay uncovered not only genes that previously had been demonstrated to be connected with genomic instability, but also identified a set of novel genes that are potentially involved in maintaining genomic stability.

## Materials and Methods

### Yeast strains and cultures

A homogeneous pool of 5083 homozygous yeast deletion strains from the Yeast Deletion Collection (Winzeler 1999) was used for all experiments described here. To avoid skewing in the strain composition of the pool due to growth rate differences between the mutant strains, the incubation times were always optimized to be as short as possible.

### Construct design and yeast bulk transformation

**Figure 1:**
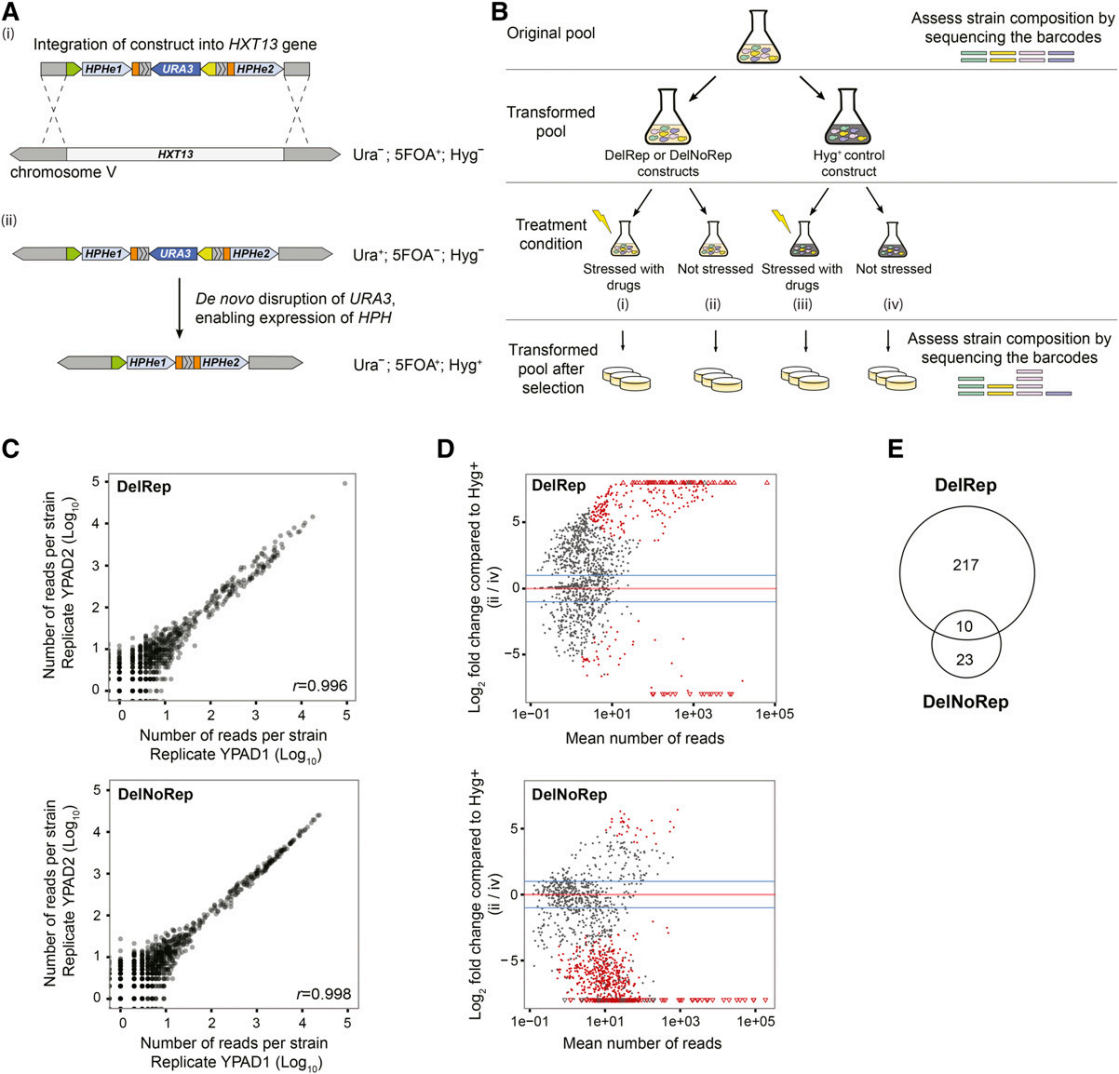
Genome-wide approach for identifying yeast knockout strains prone to acquire deletions. (A) Constructs designed to detect *de novo* deletions at large scale. (i) A cassette containing the *HPH* gene interrupted by a long intron was introduced into a nonessential region of chromosome V. Two independent versions of this construct were created, one containing direct repeats surrounding the *URA3* gene (DelRep construct) and one lacking these direct repeats (DelNoRep construct; Figure S1A in File S1). Green arrow, ADH promoter; yellow arrow, *URA3* promoter; gray striped boxes, Alu-derived direct repeat elements; and orange boxes, actin intron splice sites). (ii) Upon *de novo* deletions shortening the intron, the *HPH* gene can become spliced and hence confer hygromycin (Hyg) and 5FOA (5-fluorootic acid) resistance. A control construct with constitutive Hyg resistance was also designed (Figure S1A in File S1). (B) Experimental setup: the strain composition of the original pool from the yeast deletion collection was assessed by sequencing the barcodes of all strains. Aliquots of this pool were used to create transformed pools carrying the DelRep construct, the DelNoRep construct, or the control (Hyg+) construct. These pools were treated overnight (or 2 hr in the case of methyl methanesulfonate) with specific drugs (i and iii) or grown on rich media (ii and iv) without any stress. After treatment, strains that acquired deletions were enriched by selecting for Hyg resistance on plates. The final composition of the selected pools was again assessed by sequencing the barcodes of all recovered strains. (C) Pearson correlation between the number of reads per strain detected by sequencing the barcodes in two technical replicates transformed pools after selection, employing the DelRep and DelNoRep constructs without drug treatment {YPAD1 and YPAD2: each of the replicates grown in YPAD [Bacto-Yeast extract (10 g/L), Bacto-Peptone (20 g/L), Dextrose (20 g/L), and Adenine sulfate (0.4 g/L)] without any drug}. (D) Log2 fold changes over the mean of normalized read counts for transformed DelRep- and DelNoRep-based pools after selection [see also (ii) in (B)] relative to the Hyg+ control [see also (iv) in (B)] without drug stress applied. Strains significantly enriched at a False Discovery Rate of 10%, and with at least twofold increase, are highlighted in red. (E) Intersection of significantly enriched strains carrying the DelRep or the DelNoRep constructs.

The construct carrying the *HPH* gene described in Figure 1A was synthesized by GENEWIZ, Inc. Custom Gene Synthesis. From this construct, two other constructs were derived by performing restriction digestion and religation: one lacking the direct repeats and one showing constitutive hygromycin resistance used as a control. The constructs were linearized by restriction digestion and transformed into the pool of yeast deletion mutants. All transformations were done using the high-efficiency Lithium Acetate (LiAc), single-stranded carrier DNA and Polyethylene Glycol 3350 method (Gietz and Schiestl 1989, 2007; Knop *et al.* 1999). In summary, a 50 μl aliquot of the pooled homozygous yeast deletion collection (OD600 of 50) was inoculated into a 5 ml YPAD [Bacto-Yeast extract (10 g/L), Bacto-Peptone (20 g/L), Dextrose (20 g/L), and Adenine sulfate (0.4 g/L)] culture and incubated overnight at 30°. Then, the culture was diluted and grown again for ∼2.5 hr to reach an OD600 of 0.5–0.7. The cells were then collected and resuspended in 1 ml of water, and 100 μl with OD600 of 1 were used for transformation. To select for transformed strains, the cultures were plated on Synthetic Complete medium without uracil (Sigma-Aldrich) and left to grow for 4 d. After this, all visible colonies were collected and stored in YPAD glycerol stocks at −80°. In total, 20,000 colonies were picked for each construct transformation to cover ∼5× each ORF in the homozygous yeast deletion collection. The insertion of the construct into the *HXT13* gene (YEL069C) in chromosome V was verified by PCRs placing primers inside and outside of both sides of the construct. This locus has been used to test for chromosomal rearrangements in other studies (Chen and Kolodner 1999; Myung *et al.* 2001b), and it was shown that its disruption has no or little impact on the fitness of the cells.

### Inducing replication stress to the transformed pools of the yeast deletion collection

The transformed pools were treated overnight at 30° in YPAD cultures containing 50 μM hydroxyurea (HU), 25 μM doxorubicin (Doxo), 10 μM camptothecin (Campt), or 0.10% methyl methanesulfonate (MMS) to induce replication stress or DNA damage. A nontreated control was always included. For MMS, the treatment time was reduced to 2 hr because cell viability was lower in this drug. A total of ∼3 × 10^6^ transformed cells were used for each treatment. Following each treatment, the cells were collected, washed, and recovered by incubating them in 500 μl of YPAD for 2.5 hr at 30°. All experiments were done in triplicate.

### Selection of strains carrying rearrangements

Strains that acquired rearrangements in the constructs were selected by making dilutions of the treated and recovered cultures to a density of 6 × 10^6^ cells/ml, plating ∼2 × 10^6^ cells on hygromycin containing plates (200 μg/ml) and letting them grow at 30° for 3 d. After this, all hygromycin-resistant colonies were collected and stored at a concentration of 10^10^ cells/ml at −80° for subsequent experiments.

### Amplification and sequencing of the strain molecular barcodes

Genomic DNA purification was done using 10 μl of the hygromycin-resistant cells. The extracted DNA was used for the amplification of the unique molecular barcodes of the yeast strains by PCR using primers U1+KanB and D1+KanC for the uptags and downtags (Giaever *et al.* 2002), which amplify products of 299 and 624 bp, respectively. Both tags were amplified in a single 20 μl PCR reaction using the SequalPrep Long PCR Kit (Invitrogen). The PCR products were then purified and used for library preparation and multiplex sequencing (Smith *et al.* 2009) using the NEBNext DNA Sample Preparation kit (New England BioLabs). The sequencing was done on Illumina HiSequation 2000 or MiSeq instruments with paired ends of 101 or 150 bp, respectively.

### Strain identification

The sequencing reads were trimmed to remove the adapters and were used to detect the molecular barcodes. These barcodes were then compared to a barcode database from the *Saccharomyces* Genome Deletion Project website to identify the corresponding yeast strains. Up to three mismatches were allowed and only barcodes that could be uniquely assigned to a specific strain were kept for further analyses. The number of reads per strain was quantified and only strains supported by at least 10 sequencing reads were considered to test for enrichments.

### Identification of significantly enriched strains and gene ontology (GO) enrichment analysis

To identify significantly enriched strains that acquired deletions under a specific condition, read count data and the R package DESeq2 (Love *et al.* 2014) were used. A significant enrichment of at least twofold was required (lfcThreshold = 1). For all experiments, the differential analysis was performed using the pool of strains with constitutive hygromycin resistance as control, handled exactly the same as the treated sample. GO enrichment analysis was performed using Cytoscape (Shannon *et al.* 2003) with the BiNGO plugin (Maere *et al.* 2005). A custom reference set comprised of all strains detected in the original pool of deletion mutants was used as a background set.

### Growth rate comparison

The growth rates in rich medium of a set of strains (listed in Supplemental Material, Table S1 in File S1) with known defects in different genome maintenance pathways were compared to the growth rates of the top 10 strains detected in the enrichment assay carrying the DelRep construct and treated with drugs. Growth rate information was obtained from http://www-deletion.stanford.edu/YDPM/index.html (Steinmetz *et al.* 2002). Additionally, the growth rates of the strains detected after no stressor were also used for comparison. Wilcoxon rank-sum tests were used to assess the differences between the growth rates of these pools of strains.

### Generation and treatment of individual knockout strains

Haploid and homozygous diploid deletion strains for candidate genes were generated using a PCR deletion strategy (Baudin *et al.* 1993; Wach *et al.* 1994) on BY4741 and BY4743 backgrounds, respectively, which were transformed with the DelRep construct. Each desired ORF was substituted with a KanMX4 cassette. The newly created knockout strains carrying the DelRep construct were treated independently with the same drugs and concentrations used in the pooled screen to confirm the effect on the formation of deletions. After the treatment, strains carrying rearrangements were selected for hygromycin resistance on YPAD + Hygromycin plates and after 3 d the number of colonies formed was quantified. Experiments were done in duplicate. For each experiment, the same amounts of cells were plated. The overall number of hygromycin-resistant colonies of the BY4743 and BY4741 strains (here referred to as the wild-type controls) also transformed with the DelRep construct were compared. Additionally, a knockout strain of the nonessential and not involved in DNA repair gene *TRP5* was used as a negative control.

### Gene expression profiling of ioc4 and msh4 knockout mutants

Individual candidate knockout strains were subjected to the same enrichment experiment as described for the pooled deletion collection. Hygromycin-resistant colonies of each knockout mutant were grown under different growth conditions and harvested by centrifugation. Total RNA was isolated by bead beating and phenol-chloroform-isoamylalcohol purification. The RNA was precipitated from the upper aqueous layer and washed once with 80% ethanol. The RNA extracts were treated with RNase-free DNase I using the Turbo DNA-free kit (Ambion). RNA sequencing libraries where prepared using the TruSeq Stranded Total RNA Library Prep Kit (Illumina). Up to 20 samples were multiplexed and sequenced in one HiSeq2500 lane. Differentially expressed genes were identified by using DESeq2 (Love *et al.* 2014). Genes showing significant differences in expression in the knockout mutants compared to the wild-type strain were used to identify overrepresented groups of genes by Gene Set Enrichment Analysis (GSEA). For this, genes were ranked by log2 fold changes based on the DESeq2 output and used as input in the GSEA software (Subramanian *et al.* 2005) for the analyses with preranked gene lists.

### Data availability

RNA-sequencing (RNA-Seq) data are deposited at the European Nucleotide Archive, under the accession number PRJEB20082.

## Results and Discussion

### A high-throughput approach for identifying yeast mutant strains prone to form deletions

We set out to systematically identify yeast gene knockout strains with elevated rates of SSA deletion formation, by developing a high-throughput assay based on a set of specifically designed constructs that can confer growth advantages in the context of *de novo* deletion formation (Figure 1 and Figure S1 in File S1).

These constructs, which we stably integrated into the yeast genome through recombination at the *HXT13* locus, make use of engineered versions of the *HPH* gene conferring resistance to Hygromycin B (Gritz and Davies 1983). We separated the yeast *HPH* gene into two exons, *HPHe1* and *HPHe2*, by inserting a modified actin intron containing the *URA3* gene and its promoter between *HPHe1* and *HPHe2* (Materials and Methods). By doing so, we increased the linear distance between *HPHe1* and *HPHe2* to a length that interferes with the yeast splicing machinery (Klinz and Gallwitz 1985). We surrounded the construct with 40 bp of homologous sequences to facilitate integration into the nonessential gene *HXT13* (Chen and Kolodner 1999) on chromosome V. The additional presence of the *URA3* gene enabled positive selection (Boeke *et al.* 1984).

Using this principal setup, we generated different versions of this construct to enable investigation of patterns of deletion formation in the presence or absence of genomic DNA repeats (Figure 1Ai). The underlying principle we used is that hygromycin resistance is achieved upon formation of *de novo* deletions removing or shortening the length of the engineered *HPH* intron, to enable splicing (Figure 1Aii). One version of our construct, referred to as the DelRep construct, comprises direct homologous repeats of 30 bp derived from human DNA sequence (*i.e.*, identical *Alu*-derived DNA stretches) enabling assessment of deletion formation in the presence of homologous repeats (*e.g.*, by the SSA pathway of homologous recombination). We placed these 30 bp repeat elements in such a manner that they surrounded the *URA3* gene, allowing *URA3* disruption by repeat-mediated deletion formation. Another construct version, referred to as the DelNoRep construct, lacked these direct repeats, and hence could be employed to investigate other types of deletion formation (and concomitant *HPH* intron shortening) that may occur in the absence of homology. As a control for our experiments, we also developed a construct version exhibiting constitutive hygromycin resistance, referred to as the Hyg+ construct, which carries a short *HPH* intron that is readily spliced in yeast (Figure S1A in File S1). This control construct was used to normalize for differences in the growth rate of different knockout strains and for computing enrichment values.

Using bulk transformation in liquid culture, we introduced the constructs into a yeast pool containing all strains of the homozygous diploid yeast deletion collection (Winzeler 1999) (Figure 1B). Culture volumes and initial OD were selected to minimize experimental noise (see Materials and Methods) (Pierce *et al.* 2007). Each strain in the collection contains two individual molecular barcodes (referred to as uptags and downtags), which can be amplified by PCR and used to identify and quantify the abundance of knockout strains (Winzeler 1999). In the initial experiments, we observed high correlation between strain quantification based on uptags *vs.* downtags (Pearson correlation = 0.88; *P* < 10^−3^; Figure S1B in File S1), and hence decided to utilize uptags for strain quantification in all subsequent experiments.

Using uptags we confirmed the presence of an average of 4852 (SD ± 410) knockout strains in the original pool, representing 95.5% of all strains from the homozygous diploid yeast deletion collection. In transformed pools carrying the constructs we identified on average 76% (SD ± 1.1) of the strains from the homozygous diploid yeast deletion collection, although we recovered the majority of the strains that belong to the genome maintenance pathways (Figure S1C in File S1). We observed excellent overlap in the representation of the strains in the pools transformed with either of the three constructs (Figure S1D in File S1), suggesting equivalent transformation efficiencies for each setup.

Our genome-wide analyses using these constructs first focused on identifying strains that spontaneously acquire deletions. To this end, we selected hygromycin-resistant colonies after growth in rich media (Figure 1B). We observed Pearson correlations *r* > 0.99 when performing uptag sequencing of pools transformed with the DelRep and DelNoRep constructs, respectively, following hygromycin selection (Figure 1C). However, we generally recovered more strains with the DelRep construct than with the DelNoRep construct, identifying altogether 227 strains significantly enriched in the DelRep pool and 33 strains enriched in the DelNoRep pool relative to the Hyg+ control construct (Figure 1D). The elevated rate of barcodes identified for the DelRep construct is consistent with the notion that repeats facilitate deletion formation by mediating nonallelic homologous recombination (Eichler 1998; Iraqui *et al.* 2012) or alternatively by promoting replication fork stalling, which can also facilitate rearrangement formation (Song *et al.* 2014). This increase in deletion formation between direct repeats occurs even in the absence of any drug stress, which is consistent with naturally occurring repeats being found at recombination hotspots (Song *et al.* 2014; St. Charles and Petes 2013). In addition, solo-LTRs (the long terminal repeats at the ends of LTR-retrotransposons), which are common in yeast, have been associated with replication fork stalling and higher susceptibility to recombination-prone lesions that result in rearrangements (Song *et al.* 2014). In humans, several diseases are also caused by recurrent deletions mediated by homologous recombination between repetitive sequences (Lupski 1998; Sasaki *et al.* 2010; Yen *et al.* 1990). A relatively low number of 10 strains that were shared between the DelRep and the DelNoRep pools (Figure 1E and Table S2) point to potential differences in the underlying mechanisms protecting against deletion formation in the presence and absence of direct repeats.

### Effect of drug treatments on deletion formation

We next assessed the impact of DNA-damaging agents on the formation of deletions, reasoning that DNA damage can further elevate SV formation, which may help to increase the number of genes accessible to our genome-wide study and thus the overall resolution of our approach. Transformed pools were treated using the following chemicals in technical duplicates (Figure 1B): HU (inducing replication fork stalling; 50 μM) (Bianchi *et al.* 1986; Petermann *et al.* 2010), MMS (a DNA alkylating agent; 0.10%) (Chang *et al.* 2002), Doxo (a topoisomerase II inhibitor; 25 μM) (Patel *et al.* 1997), and Campt (a topoisomerase I inhibitor; 10 μM) (Liu *et al.* 2006). Apart from their use as mutagens, some of these drugs are employed in cancer therapy (Tan *et al.* 1973; DeBrabander *et al.* 1976; Cheung-Ong *et al.* 2013), which underscores the relevance of understanding their impact on SV formation.

**Figure 2:**
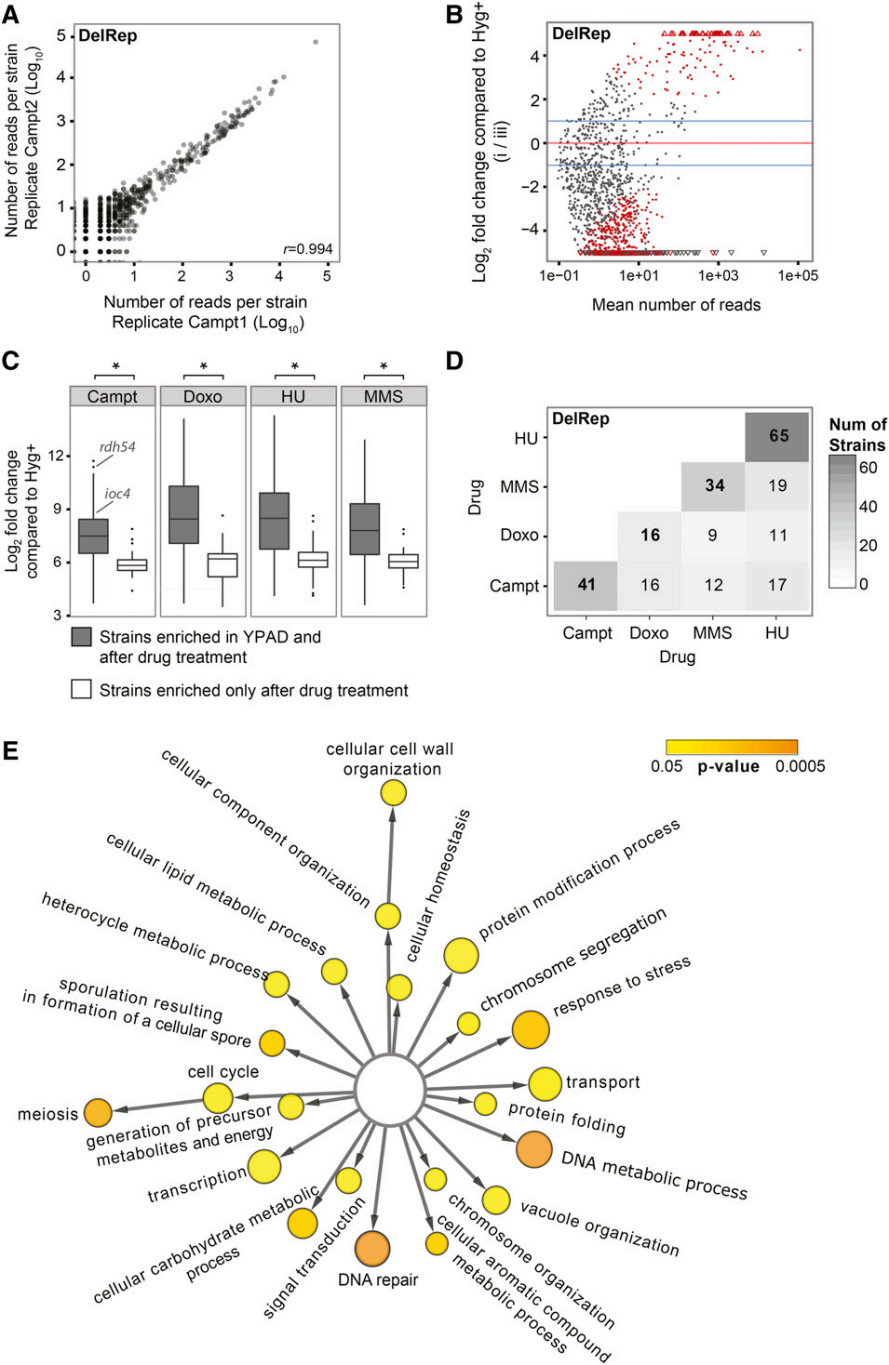
Yeast knockout strains acquire deletions between direct repeats, with and without different drug treatments. (A) Correlation between the number of reads per strain detected by sequencing the barcodes in two technical replicates for the DelRep after treatment with camptothecin (Campt) {Campt1 and Campt2: each of the replicates grown in a YPAD medium [Bacto-Yeast extract (10 g/L), Bacto-Peptone (20 g/L), Dextrose (20 g/L), and Adenine sulfate (0.4 g/L)] containing Campt}. (B) Log2 fold changes over the mean of normalized read counts for the strains transformed with the DelRep construct [(i) in (B) of Figure 1] compared to the hygromycin (Hyg+) control ((iii) in (B) from Figure 1) after treatment with Campt. Significantly enriched strains [False Discovery Rate (FDR) = 10%] with at least twofold increase are shown in red. (C) Log2 fold enrichments for strains detected after growth without treatment and after different drug treatments in strains carrying the DelRep construct (*t*-test, * *P* < 0.01). (D) Number of strains significantly enriched (FDR = 10%) that acquired deletions between direct repeats after treatment with different drugs (the total is shown in the diagonal and the number of strains shared between drug treatments is shown below the diagonal). Doxo, doxorubicin; HU, hydroxyurea; MMS, methyl methanesulfonate; and Num, number. (E) Main gene ontology terms for biological processes enriched in the strains that acquired deletion between direct repeats after drug treatment. The color represents the significance and the size the number of strains in the set that belong to each term.

We analyzed strains after overnight drug treatment (or after 2 hr in the case of MMS treatment) followed by a recovery time of 2.5 hr in YPAD, and compared results to the Hyg+ control construct. Replicates of transformed pools showed high correlation in the presence of uptags following hygromycin selection (with correlation coefficients of up to 0.999; see Figure 2A and Figure S2 in File S1). Table S3 in File S1 shows the total number of strains enriched after growth on different treatments, and following selection on hygromycin. Figure 2B depicts enriched strains grown in Campt at a False Discovery Rate (FDR) of 10% and at least twofold change compared to the control (pool transformed with Hyg+). Genes identified in this experiment include *ACE2*, a transcription factor regulating the expression of genes involved in mitosis, meiosis, and cell wall function (Doolin *et al.* 2001), as well as *ZIP2*, a meiotic gene involved in synaptonemal complex formation (Chua and Roeder 1998) (see Table S4 in File S1). Notably, *ACE2* and *ZIP2* were also enriched with other drug treatments (*e.g.*, MMS and HU, respectively; see Table S4 in File S1), indicating a general preponderance of these gene knockouts to elevate deletion formation in yeast. Similar to what is observed for spontaneously generated SVs, a higher number of strains acquired deletions between direct repeats when compared to the DelNoRep construct (*P* = 3.5 × 10^−5^; Fisher’s-exact test), in line with the facilitating role of direct repeats in SV formation. Given the relatively high number of events generated in constructs carrying direct repeats, we thus focused our following analyses on the DelRep construct, which investigate deletions formed by the SSA process (referred to as SSA-mediated deletions). We amplified the region around the construct and sequenced the PCR amplicons. We then aligned all the reads searching for split reads around the repeat regions. With this approach, we identified several reads that overlap potential breakpoints. As shown in Figure S3 in File S1, the reads aligned to either one of the direct repeats with a gap in between the repeats (indicating the deletion). This is in agreement with the SSA mechanism, whereby one of the direct repeats are kept and the other one is lost upon recombination.

Previous studies in budding yeast identified several genes that are involved in SSA processes, such as *MSH2*, *MSH3*, *RAD1*, *RAD10*, *RAD59*, and *RAD52* (Ivanov *et al.* 1996; Sugawara *et al.* 1997, 2000). In order to see if our screening approach indeed had the potential to identify deletions mediated by SSA, we investigated the underrepresented genes in our DelRep construct and reassuringly identified several of the SSA components, with the highest effects observed on *RAD10*, *RAD59*, and *MSH2*.

Interestingly, 82% (SD ± 5.1) of the strains acquiring deletions between direct repeats under drug treatments were also detected in pools grown without any stress, indicating that although drug treatment leads to overall a higher number of SSA-mediated deletions (Figure 2C), results obtained through such treatment largely hold true also in the context of spontaneous SV formation events. We observed significantly higher fold enrichment, *i.e.*, increased propensity to lead to *de novo* deletion formation, for strains identified across multiple conditions *vs.* those enriched only upon a specific treatment (*P* < 0.01; *t*-test; Figure 2C). The overlap between strains detected after treatment with different drugs was on average 45.8% (SD ± 30.1) at the given FDR threshold (Figure 2D).

### Potential roles of DNA repair, meiosis, and chromatin remodelling genes in SSA-mediated deletion formation

Genes uncovered by our approach included several genes from the DNA repair and genome maintenance pathways, including *RDH54*, *MMS2*, *IRC20*, *RAD34*, and *SHU2*. These genes increased the rate of deletions with or without drug treatment, with computed enrichment values ranging from 126- to 2048-fold depending on the treatment (see Table S5 for a complete list of strains and enrichment values). For example, *RAD34*, a gene involved in nucleotide excision repair, exhibited a 388-fold enrichment under HU treatment [Benjamini-Hochberg (BH)-adjusted *P*-value = 2.7 × 10^−5^], whereas *SHU2*, a member of the Shu complex involved in error-free postreplication repair and homologous recombination (Ball *et al.* 2009), exhibited 126-fold enrichment under HU treatment (BH-adjusted *P*-value = 6 × 10^−3^). Notably, *SHU2* has also been identified by genome-wide screening for genes suppressing gross chromosomal rearrangements (Smith *et al.* 2004), corroborating these findings.

We performed a GO analysis that revealed the GO term “DNA repair” to be among the most enriched biological processes in strains acquiring deletions (Figure 2E). However, a number of genes known to be involved in DNA repair and genome maintenance were not identified by our study, such as *SGS1* and *MRE11* (Watt *et al.* 1996; Chen and Kolodner 1999). Assessment of previously published data on growth rates for different yeast knockout strains (Steinmetz *et al.* 2002) showed that disruption of genome maintenance genes can result in severe growth defects (see Figure S4 in File S1), which may explain why some of these genes were not identified by our approach.

We next compared our results to previously published datasets. For this we used screens for chromosome loss phenotypes (Yuen *et al.* 2007), mutation-suppressing genes (Huang *et al.* 2003), loss-of-heterozygosity (LOH) (Andersen *et al.* 2008), and gross chromosomal rearrangements (Smith *et al.* 2004; Kanellis *et al.* 2007; Putnam *et al.* 2016) (Figure S5 in File S1). We identified several genes that are shared with some of these previous studies. For instance, *TSA1*, a gene involved in oxidative stress and suppression of genomic instability, was found in our screen as well as in five other studies. Additionally, *RDH54* and *MMS2*, identified in our screen with and without drug treatment, have also been identified in three of the other studies. Although none of the genes identified in our screen were found in the screen for LOH phenotypes (Andersen *et al.* 2008), suggesting that LOH does not play a role in a recombination-based deletion mechanism, we found good agreement between our candidate genes and those identified by Putnam *et al.* (2016). Our DelRep set shared 10 genes with this study (*TSA1*, *RDH54*, *MMS2*, *SHU2*, *CCS1*, *YKR023W*, *RAD30*, *UBC13*, *YKU80*, and *SNQ2*), the highest number of shared genes seen in all comparisons. Additionally, two genes from the DelNoRep set were also found to be shared with Putnam *et al.* (2016) (*HST3* and *YAP1*), highlighting the importance of our candidate genes for genomic instability.

Apart from genes that are directly involved in DNA repair, we also found that knockout of the chromatin remodeller *IOC4* resulted in a high level of deletions under different conditions, *e.g.*, with 337-fold enrichment under Campt treatment (Figure 2C and Table S5). Ioc4, together with Ioc2 and Isw1, belongs to the chromatin remodelling complex Isw1b (Vary *et al.* 2003; Maltby *et al.* 2012). *IOC4* has been previously identified in a genome-wide screen for haploinsufficient genes that might lead to genome instability in *S. cerevisiae*, although it is unclear if the effects are direct or indirect (Choy *et al.* 2013). Additionally, there is accumulating evidence of the involvement of other chromatin remodelling complexes in genome maintenance, such as for SWI/SNF chromatin remodelling complexes (Klochendler-Yeivin *et al.* 2006), which exhibit mutations in 20% of human cancers (Brownlee *et al.* 2015). Furthermore, chromatin remodelling has been implicated in regulating the access of factors involved in replication, recombination, and repair to DNA (Dion and Gasser 2013; Papamichos-Chronakis and Peterson 2013; Price and D’Andrea 2013), suggesting a potential connection between the knockout of *IOC4* and elevated rates of SSA-mediated deletions observed by our approach.

Surprisingly, following “DNA repair” and “DNA metabolic process,” the third most enriched GO term for biological processes enriched in SSA-mediated deletion-acquiring strains was “meiosis,” in spite of the fact that we performed our assays under vegetative growth conditions. Meiosis-related genes identified included *MSH4*, *ZIP2*, *SPO73*, *REC114*, and *REC107*, with enrichments ranging from 84- to 222-fold (BH-adjusted *P*-values between 0.001 and 0.02; see Figure 2E and Table S4 in File S1). Interestingly, *MSH4* and *ZIP2* are known to colocalize and form discrete foci on meiotic chromosomes (Novak *et al.* 2001). Notably, several meiosis-related knockout strains were shared between different drug treatments, including the *ZIP2* knockout strain, which we observed to be significantly enriched under Campt and HU treatments (84- and 181-fold; BH-adjusted *P*-values = 2.8 × 10^−3^ and 3 × 10^−3^, respectively). Overall, these results suggest that meiosis-related proteins frequently also assume roles in DNA damage response-related pathways during vegetative growth. In further support of this view, the human homolog of *MSH4*, *hMSH4*, in addition to its meiotic role, has recently been implicated in the maintenance of genomic stability as a suppressor of nonhomologous end joining (NHEJ)-mediated DNA repair (Her *et al.* 2003; Chu *et al.* 2013). Our results also support this notion and present evidence linking defects in *MSH4* function to the formation of deletions. Furthermore, other proteins of the same family including Msh2 and Msh3, apart from their role in mismatch repair, have been implicated in SSA-mediated deletion formation. These proteins are required for the repair of double-strand breaks between homologous sequences, and are thought to stabilize the intermediate junctions (Sugawara *et al.* 2004).

### Verification of variant formation using individual knockout strains

In order to validate the results from our genome-wide screens, and to prevent the risk of the effects observed being due to reported concerns with the yeast deletion collection, including the existence of aneuploidies or additional mutations other than the specific KO genes (Hughes *et al.* 2000; Lehner *et al.* 2007), we employed an established PCR-based gene deletion strategy (Baudin *et al.* 1993; Wach *et al.* 1994) to regenerate haploid and diploid individual yeast knockout strains for numerous candidate genes including *MSH4*, *APN2*, *ZIP2*, *IOC4*, and *ENO1*. Each individual knockout strain was transformed with the DelRep construct and subjected to the experimental workflow described in Figure 1B.

**Figure 3:**
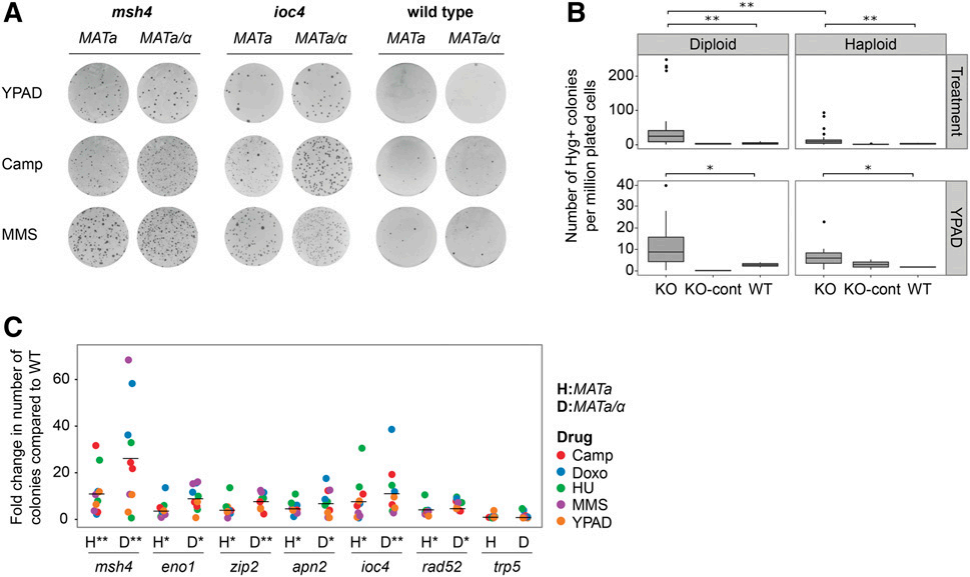
Individual KO strains confirm frequent single-strand annealing-mediated deletion formation under different drug treatments or under no stress. (A) The number of Hyg-resistant colonies was higher in the KO strains than in the WT control strain. (B) Frequency of Hyg-resistant colonies from diploid and haploid knockout strains that acquired deletions between direct repeats in the DelRep construct after growth under different drug treatments or under no stress (YPAD) (* *P* < 0.01 and ** *P* < 0.001; Wilcoxon rank-sum test). (KO_cont: Knockout control) (C) Fold increase in the number of colonies of haploid and diploid knockout strains that gained deletions compared to the WT control. KO strains were independently treated with five different drugs in duplicate experiments. Each data point shows the fold-change in the number of Hyg-resistant colonies that were recovered after treatment with the drugs compared to the WT control. Trp5 was used as a negative control. All other KO strains, in both haploid and diploid states, showed a significantly higher number of Hyg-resistant colonies compared to the WT control (* *P* < 0.01 and ** *P* < 0.001; Wilcoxon rank-sum test). Horizontal black lines mark the mean fold change. Camp, camptothecin; Doxo, doxorubicin; HU, hydroxyurea; Hyg, hygromycin; KO, knockout; MMS, methyl methanesulfonate; WT, wild-type; YAPD, Bacto-Yeast extract (10 g/L), Bacto-Peptone (20 g/L), Dextrose (20 g/L), and Adenine sulfate (0.4 g/L).

Notably, these newly created individual knockout strains exhibited higher levels of SSA-mediated deletion formation when compared to the wild-type strain (carrying the DelRep construct) and to a negative control strain (deletion of the *TRP5* gene) for both the diploid and the haploid strains (Figure 3, A and B), providing independent verification for our genome-wide approach. Rather strikingly, the *msh4* knockout strain showed the highest increase in the number of resistant colonies in both haploid and diploid strains. This increase was even higher than for the knockout of *RAD52*, an essential gene for SSA, which we employed as a positive control. This result was unexpected given the fundamental role of Rad52 in SSA-mediated DNA repair (Sugawara and Haber 1992). Whether this increase in the number of events was due to an increased number of events repaired in an SSA-independent manner by the NHEJ pathway requires further investigation. Furthermore, the knockout of *IOC4* also resulted in a marked increase of deletion formation (Figure 3, A and C), corroborating our findings based on the genome-wide assay.

Our experiments further revealed higher deletion rates in diploids than in the corresponding haploid strains in the presence of drug stress (*P* < 0.001; Wilcoxon rank-sum test), except for the wild-type and negative control strains transformed with the DelRep construct (Figure 3B), indicating an influence of ploidy on deletion generation. Indeed, yeast cells were previously shown to tolerate higher numbers of mutations in a diploid context (Mable and Otto 2001; Anderson *et al.* 2004; Lada *et al.* 2013) and to exhibit increased genomic instability in polyploid cells (Mayer *et al.* 1992; Storchova 2014; Selmecki *et al.* 2015) [similar to what also has been observed in mammals (Fujiwara *et al.* 2005) (Mardin *et al.* 2015)], likely because of reduced fitness effects in a heterozygous state. We further observed that most knockout strains acquired deletions even in the absence of drug stress, consistent with a strong genotype effect (Figure 3C).

### Altered gene expression signatures in response to SSA-mediated deletion formation by ioc4 and msh4

**Figure 4:**
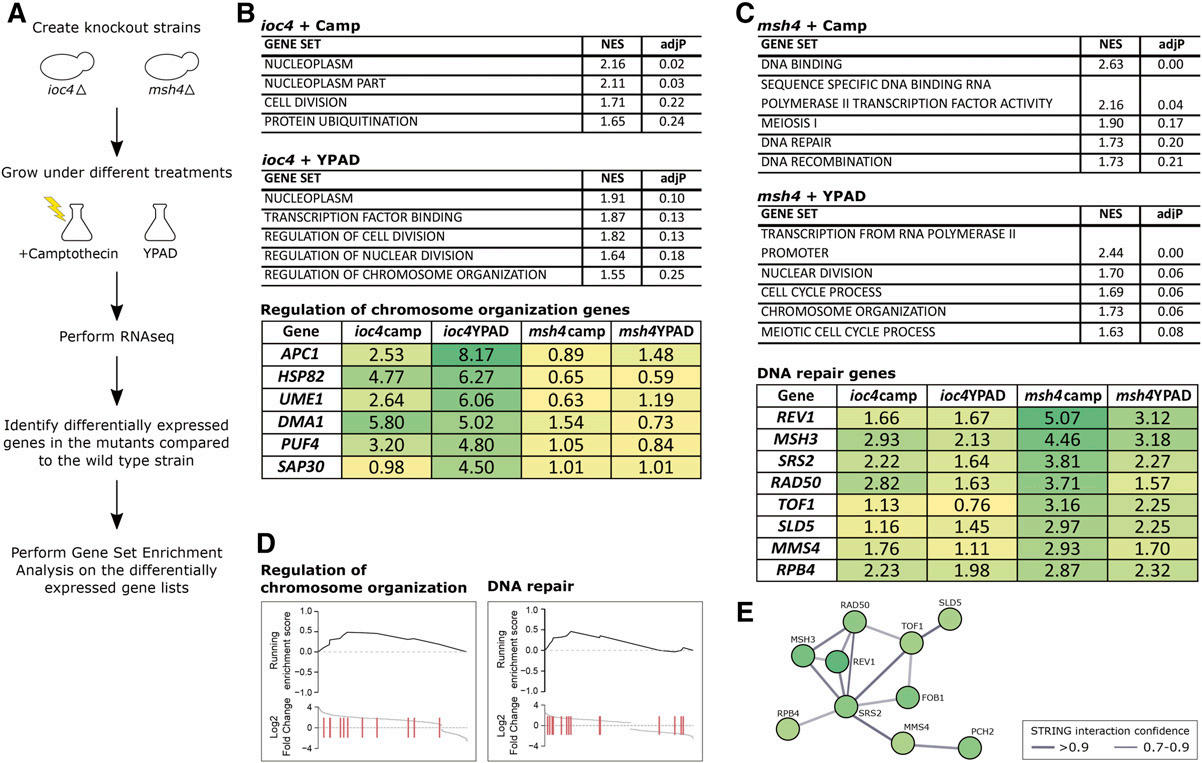
Gene expression profiling reveals different gene sets enriched in *ioc4* and *msh4* knockout strains. (A) Experimental setup for gene expression profiling. Three technical replicates were sequenced for each knockout and wild-type strain. (B) Top significantly enriched gene sets (derived from Gene Set Enrichment Analysis) in *ioc4* knockout strain grown in the presence and absence of Camp. The matrix shows the DESeq2 Log2 fold changes for the genes belonging to the “Regulation of chromosome organization” gene set in each knockout strain and treatment (enrichments compared to the Hyg+ control). (C) Similar to (B), but for *msh4* knockout strain. (D) Enrichment scores for the genes belonging to “Regulation of chromosome organization” and “DNA repair” gene sets. (E) Search Tool for Recurring Instances of Neighbouring Genes (STRING) interactions for the “DNA repair” gene set. adjP, Benjamini–Hochberg-adjusted *P*-value; Camp, camptothecin; Hyg, hygromycin; NES, Normalized Enrichment Score; RNAseq, RNA sequencing; YAPD, Bacto-Yeast extract (10 g/L), Bacto-Peptone (20 g/L), Dextrose (20 g/L), and Adenine sulfate (0.4 g/L).

Genes involved in meiosis and chromatin remodelling identified by our study currently lack direct molecular evidence concerning roles in genomic stability maintenance and SV formation. As an initial step toward uncovering their actual roles in this context, we investigated how *msh4* and *ioc4* gene knockouts affect gene expression profiles. We thus performed total mRNA sequencing (RNA-Seq) on *msh4* and *ioc4* knockout strains as well as the wild-type strain grown in the absence of drug stress or subjected to Campt treatment (Figure 4A). We sequenced three technical replicates for each strain to an average of 9.3 million reads, used DESeq2 (Love *et al.* 2014) for identification of differentially expressed genes, and subsequently performed GSEA (Subramanian *et al.* 2005) to identify groups of differentially expressed genes.

Both knockout strains showed enrichment of diverse groups of differentially expressed genes. Knockout of *IOC4* resulted in differential expression of genes related to cell division and chromosome organization (Figure 4, B and D), indicating a relationship of this chromatin remodelling gene with these processes. Within these categories, genes such as *APC1* (coding for a subunit of the anaphase promoting complex) (Zachariae *et al.* 1996), *DMA1* (important for proper mitotic spindle positioning), and *HOS1* (coding for a histone deacetylase) were found to be overexpressed in *ioc4* knockout strain when grown with or without drug stress. Additionally, we observed several transcription factor-binding proteins to be overexpressed in the *ioc4* knockout strain, including *SWI1* (a member of the SWI/SNF chromatin remodelling complex) (Hirschhorn *et al.* 1992), which notably has previously been implicated in genome maintenance (Klochendler-Yeivin *et al.* 2006). The enrichment of gene sets related to chromosome organization suggests that the knockout of *IOC4*, and the likely abrogation of the activity of its complex Isw1b, results in an upregulation of genes involved in chromosome organization (such as *SWI1* and *CYC8*).

For the *msh4* knockout strain grown in the presence of Campt, genes related to DNA binding, repair, and recombination (including the *MMS4*, *RAD50*, and *TOF1* genes) were significantly overexpressed relative to the wild-type strain (Figure 4, C–E). The overexpression of these genes suggests that, in the absence of the *MSH4* gene and the simultaneous presence of DNA damage, increased levels of DNA repair genes may help cells cope with the absence of *MSH4*.

In order to investigate the potential link between overexpressed genes and genomic instability, we obtained a list of 245 dosage chromosome instability genes (dCIN) in yeast, which when overexpressed cause chromosome instability (Duffy *et al.* 2016). We then overlapped the list of overexpressed genes in our experiments to this dCIN gene list (Table S6 in File S1). We looked at the genes significantly overexpressed in the *ioc4* or *msh4* deletion mutants grown in the presence or absence of Campt and that have a dCIN effect. Interestingly, among the genes that have been reported to have a dCIN effect, we detected *DMA1*, which is one of the genes belonging to the “Chromosome organization” gene set found to be overrepresented in the *ioc4* mutant (Figure 4B). Similarly, *SRS2* also has been reported to be a dCIN gene, and it belongs to the “DNA repair” gene set, also shown to be overrepresented in the *msh4* deletion mutant (Figure 4C). In summary, our results highlight two important gene sets that are activated in response to deletion formation by *IOC4* or *MSH4* gene knockouts and may directly or indirectly affect genome instability.

In this study, we present a genome-wide assay to identify genes that, when knocked out, are prone to deletion formation. To this end, we employed two different constructs, one of which we termed DelRep, which investigates the contributors to SSA-mediated deletions. With another construct that we named DelNoRep, we also analyzed other types of deletion formation mechanisms; however, we found that the SSA-mediated deletion mechanisms occur much more frequently in our experimental system.

Effects of gene knockouts can be exacerbated under stress conditions induced by chemical agents, facilitating our approach. Our genome-wide study implicates chromatin remodelling and meiosis genes as novel factors with unexpected roles in the maintenance of genome integrity. These results may pave the way for further functional studies aimed at fully understanding the functional impact of these candidate genes at a genome-wide level and the precise mechanisms by which they preserve genomic stability.

## Supplementary Material

Supplemental material is available online at www.g3journal.org/lookup/suppl/doi:10.1534/g3.117.300165/-/DC1.
