# Supplementary material for: Systematic Identification of Determinants for Single-Strand Annealing-Mediated Deletion Formation in Saccharomyces cerevisiae
Source: G3 (Bethesda). 2017 Aug 17;7(10):3269–79. doi: 10.1534/g3.117.300165 (PMC5633378; doi:10.1534/g3.117.300165)
Supplement: Supplementary file 3 [file 3269FileS1.docx]

**Supplemental Material**

**Segura-Wang et al.**


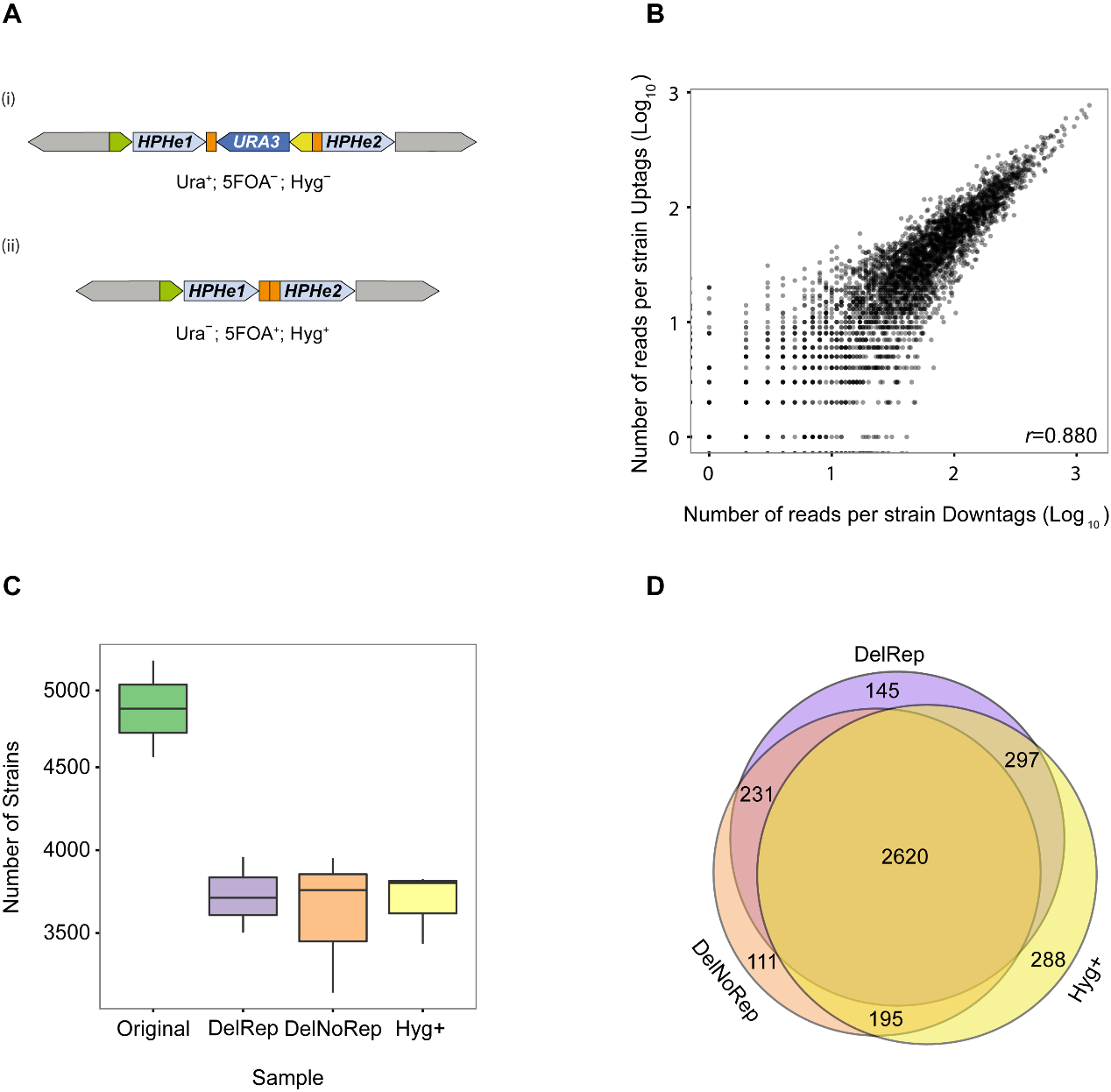


**Figure S1. The yeast deletion collection transformed with the DelRep and DelNoRep constructs has a good representation of the original pool of yeast knock-out strains.**

(A) Constructs designed to detect de novo deletions at large scale. (i) The DelNoRep version of the construct lacks direct repeats surrounding the *URA3* gene. (ii) A Hyg+ control construct lacks the *URA3* gene and has constitutive hygromycin resistance. (Green arrow: ADH promoter, Yellow arrow: URA3 promoter, Orange boxes: actin intron splice sites). (B) Correlation between the number of reads per strain detected by sequencing the downtags and the uptags (Pearson correlation coefficient is shown, *p<*0.001). (C) Number of strains detected by sequencing the uptags of the original pool of yeast knock-out mutants, and after transforming the DelRep, DelNoRep and Hyg+ constructs into the pool. Three aliquots of the original and transformed pools were used for the sequencing and identification of the strains. (D) Number of strains shared between the pools of strains transformed with different constructs.


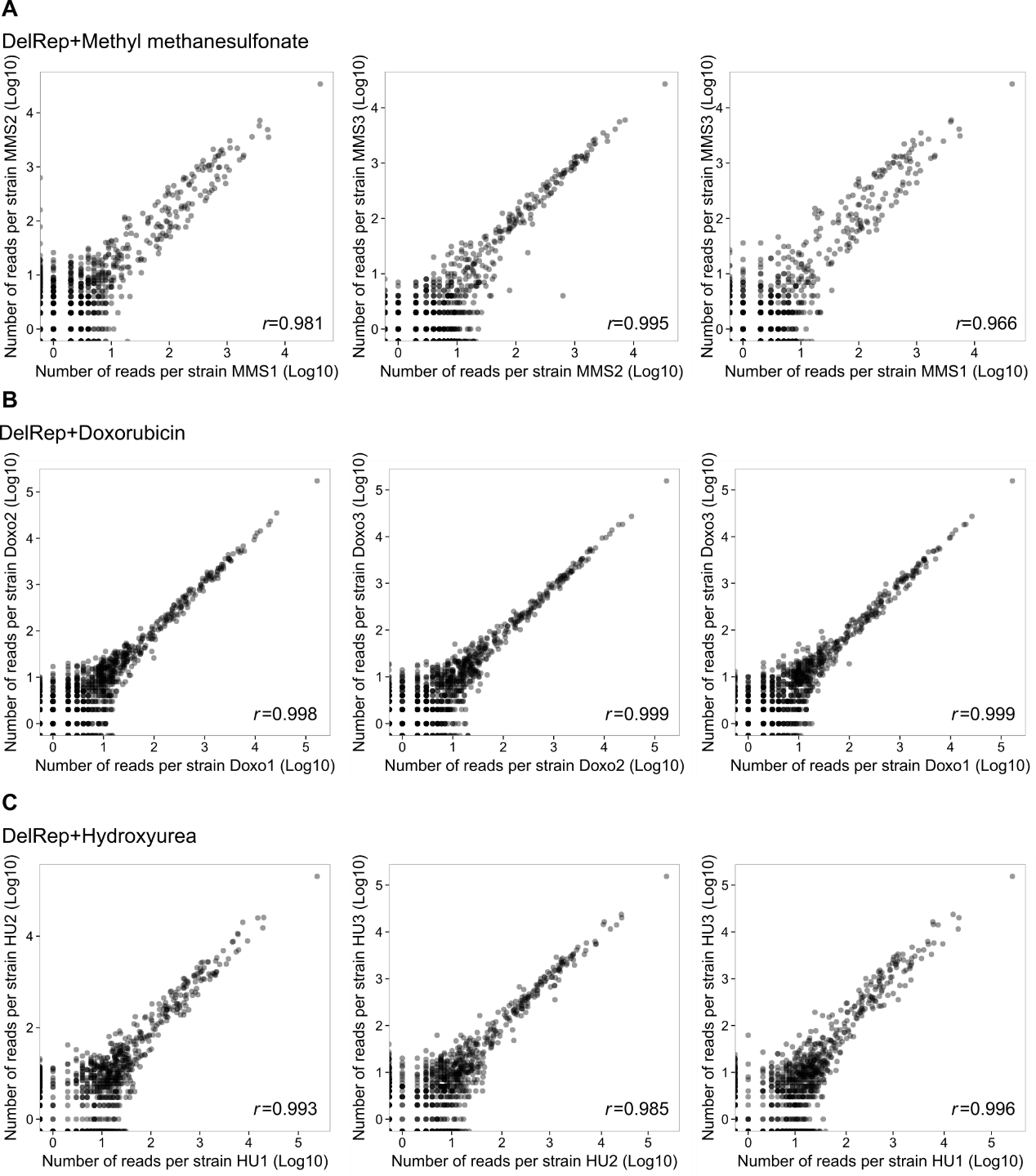


**Figure S2.** **The correlation between technical replicates was very high.**

Correlation between the number of reads per strain detected by sequencing the barcodes of three technical replicates in yeast deletion collection pools transformed with the DelRep construct after treatment with (A) MMS, (B) Doxorubicin or (C) Hydroxyurea. Pearson correlation coefficients are shown (*p*<0.001).


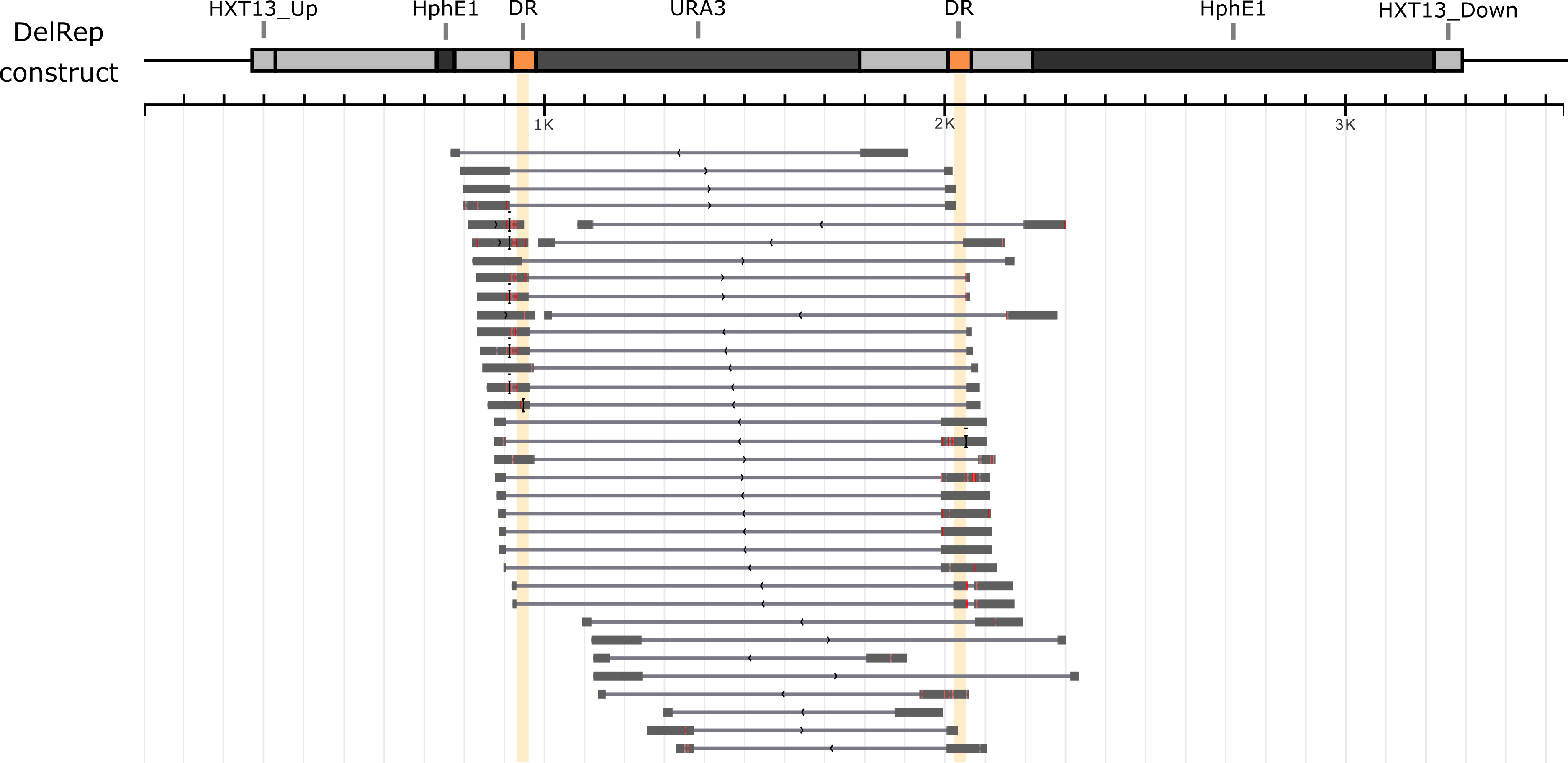


**Figure S3. Examples of sequencing reads overlapping potential deletion events in the DelRep construct.**

Potential deletions (indicated by the gap between the direct repeats) in the DelRep construct detected by split-reads alignments around the direct repeat regions.


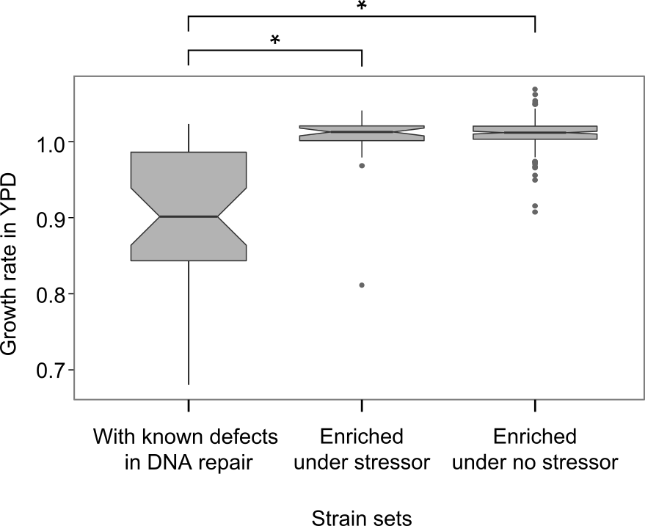


**Figure S4. Yeast knock-out strains with defects in genome maintenance pathways have a significantly lower growth rate.**

Yeast strains identified with our whole-genome assay, either enriched under the influence of a drug or those enriched when grown in rich media without drugs, showed significantly higher growth rates than a set of yeast knock-out strains with known defects in DNA repair (the list of strains is shown in the Supplemental Experimental Procedures) (*: *p*<0.0001).


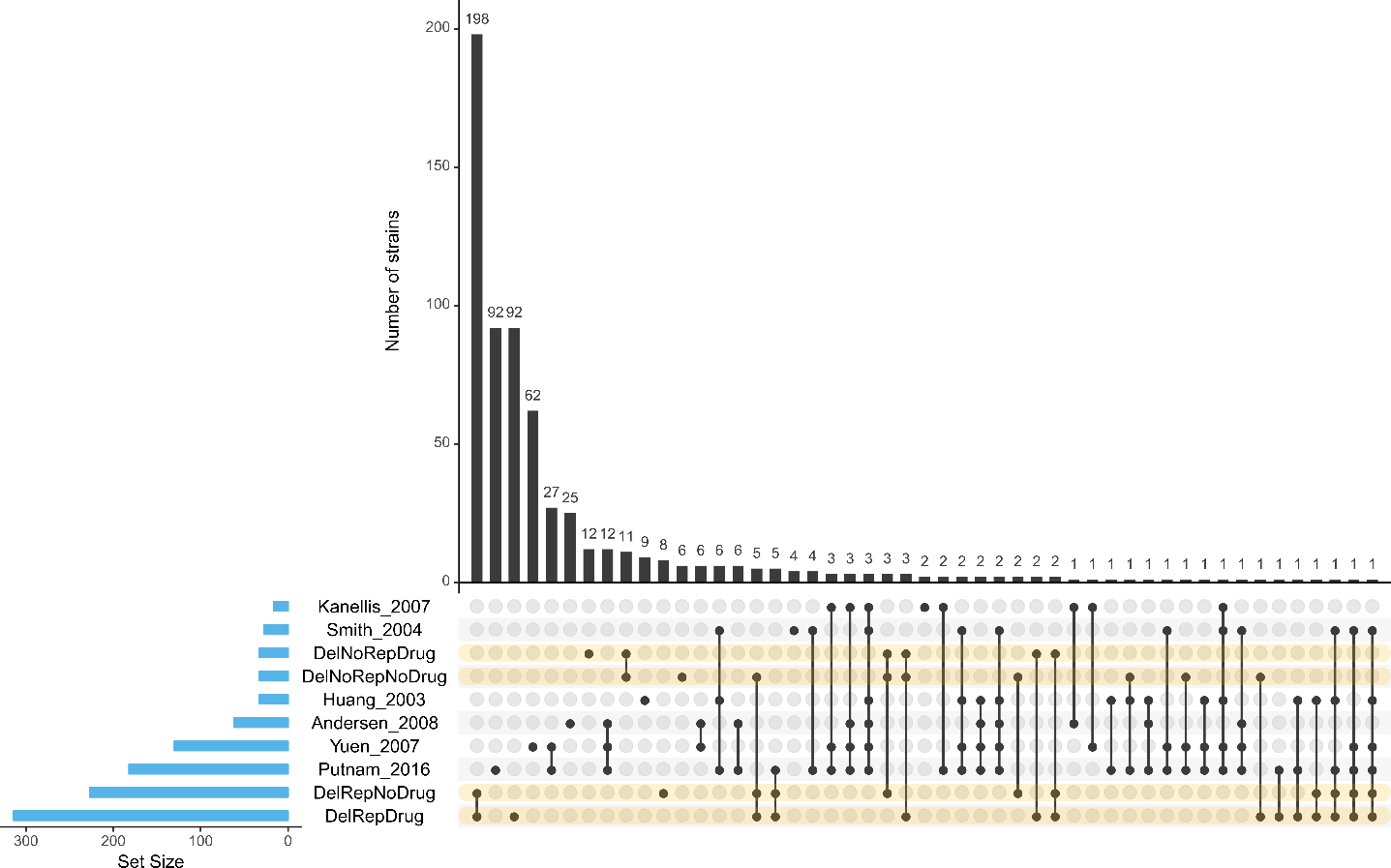


**Figure S5. Comparison of enriched strains carrying the DelRep and DelNoRep constructs with published lists of genes involved in genomic instability.**

Shared strains enriched in the DelRep and DelNoRep pools (in the presence and absence of a drug, classified all in DelRepDrug, DelRepNoDrug, DelNoRepDrug and DelNoRepNoDrug, respectively) and several other lists of genes involved in genomic instability. Data from (Huang *et al.*, 2003; Smith *et al.*, 2004; Kanellis *et al.*, 2007; Yuen *et al.*, 2007; Andersen *et al.*, 2008; Putnam *et al.*, 2016).

**Table S1.** Yeast knock-out strains with known defects in DNA repair, DNA recombination and chromosome segregation.

| **Deleted gene** | **General Function** |
| --- | --- |
| *MRE11* | DSB repair by recombination |
| *RAD52* | DSB repair by recombination |
| *SGS1* | Illegitimate recombination suppression |
| *RAD59* | Recombination, single-strand annealing |
| *ESC2* | Transcriptional Silencing, Recombination, DNA Damage Checkpoint |
| *MSH2* | Mismatch repair |
| *LIF1* | Non-homologous end-joining |
| *SAE2* | Hairpin DNA processing |
| *RTT107* | Replication fork repair |
| *SRS2* | DNA repair, genome stability |
| *RAD50* | DSB repair, NHEJ |
| *MMS4* | Recombination and repair |
| *RAD6* | Histone Modifying, DSB repair, Checkpoint Control |
| *PMS1* | Mismatch Repair |
| *RAD10* | NER (Nucleotide Excision Repair), DSB repair |
| *MUS81* | Replication fork restart, DNA repair |
| *RAD18* | Post-replication repair, Ubiquitin ligase |
| *CHK1* | Chromosome Segregation (DNA damage checkpoint) |
| *SGO1* | Chromosome Segregation, Spindle Checkpoint |
| *CTF19* | Chromosome Segregation |
| *CIN8* | Chromosome Segregation |
| *NUP170* | Chromosome Segregation, Nuclear Pore Complex |
| *RSC1* | Chromatin Remodelling |
| *ASF1* | Chromatin Remodelling, Ty1 Transposition |
| *ISW1* | Chromatin Remodelling |
| *SPT2* | Chromatin Remodelling |
| *SWR1* | Chromatin Remodelling |
| *CTF8* | DNA damage replication checkpoint, Sister Chromatid Cohesion |
| *CSM3* | DNA damage replication checkpoint, meiotic chromosome segregation |
| *TOF1* | DNA replication checkpoint |
| *RAD27* | Okazaki fragment processing |
| *POL32* | Error-prone DNA synthesis |
| *RRM3* | Ty1 Transposition, Replication Fork Stalling |
| *CTF4* | DNA Replication, Sister Chromatid Cohesion |
| *SLX1* | Replication fork restart |
| *DUN1* | DNA damage replication checkpoint, post-replicative DNA repair |
| *HST3* | Transcriptional Silencing |
| *ARD1* | Transcriptional Silencing, N-terminal acetylation |
| *RAD24* | DNA damage checkpoint signalling |
| *DOT1* | Histone Modifying, DNA damage response |
| *SET1* | Histone Modifying |
| *PMR1* | Protein sorting |
| *YKU70* | Telomere maintenance and NHEJ |
| *YOL086W-A* | Genome stability maintenance, homolog of Fanconi Anaemia Complex |
| *GCN5* | Transcription regulation |

**Table S2.** Knock-out strains transformed with the DelRep or the DelNoRep constructs enriched after growth on rich media, *i.e.* in the absence of drugs. Attached as Excel file.

**Table S3.** Total number of strains significantly enriched after growth on different treatment conditions and selection for hygromycin resistance for the yeast pools transformed with the DelRep or the DelNoRep constructs. The number of unique strains, *i.e.* not present in the YPAD control, that were detected per treatment is shown in parenthesis.

|  | **Construct** | |
| --- | --- | --- |
| **Treatment** | **DelRep** | **DelNoRep** |
| Campt | 231 (41) | 12 (6) |
| Doxo | 256 (16) | 12 (4) |
| MMS | 210 (34) | 21 (9) |
| HU | 262 (65) | 4 (1) |
| YPAD (control) | 227 | 33 |

**Table S4.** Top ten significantly enriched strains in the pools transformed with the DelRep construct after different drug treatments. Strains selected for experimental validations are shown in bold. (Gene Name indicates the particular gene that is deleted in each strain. padj: Benjamini- Hochberg adjusted *p*-value. 11 strains are shown for HU to include *REC114*).

| **Strain** | **Fold Change** | **padj** | **Gene Name** | **Function** |
| --- | --- | --- | --- | --- |
| **Campt** | | | | |
| YFL003C | 7.86 | 0.001 | ***MSH4*** | Protein involved in meiotic recombination; required for normal levels of crossing over, colocalizes with Zip2p to discrete foci on meiotic chromosomes |
| YGL226C-A | 7.28 | 0.004 | *OST5* | Zeta subunit of the oligosaccharyltransferase complex of the ER lumen |
| YGL248W | 7.05 | 0.006 | *PDE1* | Low-affinity cyclic AMP phosphodiesterase |
| YGR254W | 6.64 | 0.018 | ***ENO1*** | Enolase I, a phosphopyruvate hydratase |
| YDR370C | 6.51 | 0.013 | *DXO1* | mRNA 5'-end-capping quality-control protein |
| YER046W | 6.44 | 0.02 | ***SPO73*** | Meiosis-specific protein of unknown function |
| YGL249W | 6.41 | 0.028 | ***ZIP2*** | Meiosis-specific protein; involved in normal synaptonemal complex formation and pairing between homologous chromosomes during meiosis |
| YBR169C | 6.3 | 0.025 | *SSE2* | Member of the heat shock protein 70 (HSP70) family |
| YGR100W | 6.21 | 0.049 | *MDR1* | Cytoplasmic GTPase-activating protein |
| YLR131C | 6.2 | 0.062 | ***ACE2*** | Transcription factor required for septum destruction after cytokinesis |
| **Doxo** |  |  |  |  |
| YBR217W | 8.62 | 1.27E-04 | *ATG12* | Ubiquitin-like modifier involved in autophagy and the Cvt pathway |
| YER046W | 7.67 | 0.002 | ***SPO73*** | Meiosis-specific protein of unknown function |
| YGR015C | 7.54 | 0.002 | *-* | Putative protein of unknown function |
| YBR169C | 7.39 | 0.005 | *SSE2* | Member of the heat shock protein 70 (HSP70) family |
| YDL093W | 7.15 | 0.005 | *PMT5* | Protein O-mannosyltransferase |
| YDR312W | 7.12 | 0.008 | *SSF2* | Protein required for ribosomal large subunit maturation |
| YDR503C | 7.06 | 0.009 | *LPP1* | Lipid phosphate phosphatase |
| YGR100W | 7.02 | 0.009 | *MDR1* | Cytoplasmic GTPase-activating protein |
| YOL158C | 7 | 0.007 | *ENB1* | Endosomal ferric enterobactin transporter |
| YBL055C | 6.9 | 0.011 | *-* | 3'-5' exonuclease and endonuclease with a possible role in apoptosis |
| **HU** |  |  |  |  |
| YDR314C | 8.6 | 2.70E-05 | ***RAD34*** | Protein involved in nucleotide excision repair (NER) |
| YJR082C | 8.3 | 4.49E-05 | *EAF6* | Subunit of the NuA4 acetyltransferase complex |
| YJL171C | 7.8 | 0.001 | *-* | GPI-anchored cell wall protein of unknown function |
| YGL249W | 7.48 | 0.003 | ***ZIP2*** | Meiosis-specific protein; involved in normal synaptonemal complex formation and pairing between homologous chromosomes during meiosis |
| YBL019W | 7.19 | 0.003 | ***APN2*** | Class II abasic (AP) endonuclease involved in repair of DNA damage |
| YBR233W | 7.17 | 0.004 | *PBP2* | RNA binding protein; involved in the regulation of telomere position effect and telomere length |
| YDL110C | 7.04 | 0.004 | *TMA17* | ATPase dedicated chaperone that adapts proteasome assembly to stress |
| YDR078C | 6.98 | 0.006 | ***SHU2*** | Component of the Shu complex, which promotes error-free DNA repair |
| YDR421W | 6.97 | 0.014 | *ARO80* | Zinc finger transcriptional activator of the Zn2Cys6 family |
| YGR238C | 6.96 | 0.01 | *KEL2* | Protein that negatively regulates mitotic exit |
| YMR133W | 6.94 | 0.013 | ***REC114*** | Protein involved in early stages of meiotic recombination |
| **MMS** |  |  |  |  |
| YDR497C | 14.13 | 0.001 | *ITR1* | Myo-inositol transporter; member of the sugar transporter superfamily |
| YLR047C | 17.61 | 0.002 | *FRE8* | Protein with sequence similarity to iron/copper reductase |
| YLR131C | 4.46 | 0.015 | ***ACE2*** | Transcription factor required for septum destruction after cytokinesis |
| YJL083W | 3.3 | 0.028 | *TAX4* | EH domain-containing protein |
| YKL061W | 3.9 | 0.037 | *BLI1* | Subunit of the BLOC-1 complex involved in endosomal maturation |
| YBR169C | 2.62 | 0.025 | *SSE2* | Member of the heat shock protein 70 (HSP70) family |
| YBL052C | 4.13 | 0.029 | *SAS3* | Histone acetyltransferase catalytic subunit of NuA3 complex |
| YGL257C | 5.74 | 0.025 | *MNT2* | Mannosyltransferase |
| YLR246W | 3.21 | 0.051 | *ERF2* | Subunit of a palmitoyltransferase |
| YLR456W | 7.83 | 0.025 | *-* | Putative pyridoxal 5'-phosphate synthase |

**Table S5.** Strains transformed with the DelRep construct that show the highest fold enrichments after growth with and without drug treatment. In bold, genes that are related to DNA repair and genome maintenance pathways. Genes uniquely detected in each drug are underlined. Attached as Excel file.

**Table S6.** Significantly overexpressed genes in the ioc4 or msh4 knock-out mutants (in the presence or absence of camptothecin) that have a dosage CIN effect (i.e. genes that when overexpressed lead to increased chromosome instability). dCIN gene list obtained from Duffy et al. (2016).

|  | **Gene** | **dCIN**  **(Duffy_2016)** | **Ioc4**  **Camp** | **Ioc4**  **YPAD** | **Msh4**  **Camp** | **Msh4**  **YPAD** |
| --- | --- | --- | --- | --- | --- | --- |
| 1 | *YDR124W* | 1 | 1 | 1 | 1 | 1 |
| 2 | *YDR248C* | 1 | 1 | 1 | 1 | 0 |
| 3 | *YFL064C* | 1 | 1 | 1 | 1 | 0 |
| 4 | *BNI4* | 1 | 1 | 1 | 0 | 0 |
| **5** | ***DMA1*** | **1** | **1** | **1** | **0** | **0** |
| 6 | *ECL1* | 1 | 1 | 1 | 0 | 0 |
| 7 | *ENV9* | 1 | 1 | 1 | 0 | 0 |
| 8 | *HOS4* | 1 | 1 | 1 | 0 | 0 |
| 9 | *RLM1* | 1 | 1 | 1 | 0 | 0 |
| 10 | *RNA15* | 1 | 1 | 1 | 0 | 0 |
| 11 | *CDC13* | 1 | 1 | 0 | 0 | 0 |
| 12 | *AFT1* | 1 | 0 | 1 | 0 | 0 |
| 13 | *CBF2* | 1 | 0 | 1 | 0 | 0 |
| 14 | *CDC4* | 1 | 0 | 1 | 0 | 0 |
| 15 | *CPR5* | 1 | 0 | 1 | 0 | 0 |
| 16 | *DNA2* | 1 | 0 | 1 | 0 | 0 |
| 17 | *GIP3* | 1 | 0 | 1 | 0 | 0 |
| 18 | *KIN3* | 1 | 0 | 1 | 0 | 0 |
| 19 | *MKK2* | 1 | 0 | 1 | 0 | 0 |
| 20 | *NDT80* | 1 | 0 | 1 | 0 | 0 |
| 21 | *NTG2* | 1 | 0 | 1 | 0 | 0 |
| 22 | *NUP100* | 1 | 0 | 1 | 0 | 0 |
| 23 | *PHD1* | 1 | 0 | 1 | 0 | 0 |
| 24 | *SAE2* | 1 | 0 | 1 | 0 | 0 |
| 25 | *VTS1* | 1 | 0 | 1 | 0 | 0 |
| 26 | *ITC1* | 1 | 0 | 0 | 1 | 1 |
| 27 | *ADY4* | 1 | 0 | 0 | 1 | 0 |
| 28 | *CAT8* | 1 | 0 | 0 | 1 | 0 |
| 29 | *ELP2* | 1 | 0 | 0 | 1 | 0 |
| 30 | *FOB1* | 1 | 0 | 0 | 1 | 0 |
| 31 | *NUP53* | 1 | 0 | 0 | 1 | 0 |
| **32** | ***SRS2*** | **1** | **0** | **0** | **1** | **0** |
